# Mouse models of atherosclerosis and their suitability for the study of myocardial infarction

**DOI:** 10.1007/s00395-020-00829-5

**Published:** 2020-11-30

**Authors:** Pelin Golforoush, Derek M. Yellon, Sean M. Davidson

**Affiliations:** The Hatter Cardiovascular Institute, 67 Chenies Mews, London, WC1E 6HX UK

**Keywords:** Atherosclerosis, Ischaemia, Reperfusion, Cardioprotection, Myocardial infarction, Mice, Coronary artery, Vascular function

## Abstract

Atherosclerotic plaques impair vascular function and can lead to arterial obstruction and tissue ischaemia. Rupture of an atherosclerotic plaque within a coronary artery can result in an acute myocardial infarction, which is responsible for significant morbidity and mortality worldwide. Prompt reperfusion can salvage some of the ischaemic territory, but ischaemia and reperfusion (IR) still causes substantial injury and is, therefore, a therapeutic target for further infarct limitation. Numerous cardioprotective strategies have been identified that can limit IR injury in animal models, but none have yet been translated effectively to patients. This disconnect prompts an urgent re-examination of the experimental models used to study IR. Since coronary atherosclerosis is the most prevalent morbidity in this patient population, and impairs coronary vessel function, it is potentially a major confounder in cardioprotective studies. Surprisingly, most studies suggest that atherosclerosis does not have a major impact on cardioprotection in mouse models. However, a major limitation of atherosclerotic animal models is that the plaques usually manifest in the aorta and proximal great vessels, and rarely in the coronary vessels. In this review, we examine the commonly used mouse models of atherosclerosis and their effect on coronary artery function and infarct size. We conclude that none of the commonly used strains of mice are ideal for this purpose; however, more recently developed mouse models of atherosclerosis fulfil the requirement for coronary artery lesions, plaque rupture and lipoprotein patterns resembling the human profile, and may enable the identification of therapeutic interventions more applicable in the clinical setting.

## Introduction

### Myocardial infarction and protection from ischaemia/reperfusion injury

Cardiovascular disease (CVD) is the leading cause of mortality worldwide, accounting for an estimated 17.9 million deaths annually, representing 31% of all global deaths [149]. Four out of five CVD associated deaths are caused by myocardial infarction (MI) and ischaemic stroke [149]. These figures show there is an immediate need for appropriate interventions to improve survival and prognosis for CVD patients.

MI is caused by obstruction of blood flow through one of the major coronary arteries supplying the myocardium, usually due to atherosclerotic plaque rupture and thrombosis. Prolonged ischaemia results in oncotic, necrotic, apoptotic and necroptotic death of heart muscle [31, 45, 96, 103]. The extent of cell death (infarct size) depends crucially on the duration of ischaemia, the size of the ischaemic area or area at risk (AAR), the degree of coronary collateral blood flow and the extent of coronary microvascular dysfunction [14, 71]. In order to salvage the ischaemic myocardium at risk of death, the tissue must be reperfused as soon as possible. Current strategies of intervention for acute ST-segment–elevation MI (STEMI) are based on opening occluded arteries using percutaneous coronary intervention or thrombolysis, or bypassing the occluded arteries by coronary artery bypass surgery (CABG). Nonetheless, reperfusion itself triggers several damaging processes leading to myocardial injury due to oxidative stress, cytosolic and mitochondrial calcium overload, rapid restoration of intracellular pH, opening of the mitochondrial permeability transition pore (MPTP) leading to the release of cytochrome c and induction of apoptosis, gap junction changes and inflammation [71]. Mitochondrial outer membrane permeabilisation (MOMP) leads to the re-localisation of cytochrome c from mitochondria to the cytosol, where it initiates apoptosome formation and caspase activation [151]. In addition, reperfusion leads to vascular injury including impaired vasomotion and capillary destruction which contribute to microvascular obstruction (MVO) and lack of reflow [74]. Consequently, the attempt to salvage the myocardium can, itself, cause a degree of irreversible injury and cell death, which is referred to as reperfusion injury.

Extensive studies have been performed over the past 30 + years to develop strategies to limit ischaemia and reperfusion (IR) injury [68, 69]. Although many have been identified that are cardioprotective in animal models, none have yet been translated effectively to patients. One of the first cardioprotective drug cocktails investigated, glucose-insulin-potassium therapy, was found to protect in animals studies, but in clinical trials of totalling over 27,000 patients with acute coronary syndrome, no convincing benefit was seen [92] (reviewed in [100]). A phase III trial of cariporide, an inhibitor of the sodium hydrogen exchanger isoform-1 (NHE-1), in patients undergoing coronary artery bypass graft surgery, found a highly significant reduction in myocardial injury, although this was unfortunately accompanied by a significant increase in cerebrovascular events and mortality [132]. More recently, remote ischaemic conditioning (RIC), which is clearly effective in animal models [22], had no benefit on either myocardial infarct size or clinical outcomes at 12 months in the multi-centre CONDI-2/ERIC-PPCI trial of 5401 STEMI patients treated with PPCI [70]. One class of agents that has shown some promise in clinical trials are the glucagon-like-peptide-1 analogues such as exenatide and liraglutide [26, 122, 222], although so far they have only been studied in small, proof-of-concept trials.

Possible reasons for the failures of translation described above have been extensively discussed [68, 69, 76]. One issue that has been widely discussed is the fact that most rodent studies of MI are performed in healthy, young animals, whereas the typical cardiac patient is elderly, and may exhibit complex multi-morbidity [19, 36, 176, 202]. Attempts have been made to overcome this issue by using models of hypertension, hyperlipidaemia, diabetes, age etc., and indeed, these have been seen to have an effect on the signalling pathways involved in cardioprotection [6, 43, 119, 120, 162, 218]. This has led to the proposal that multitarget strategies will be required to effectively reduce IR injury in patients [32]. Clearly, however, the most prevalent morbidity in the STEMI patient population is coronary atherosclerosis. Angiographic studies find evidence of significant obstructive coronary artery disease in ~ 95% of MI patients [33, 34, 155]. Surprisingly few IR studies have been performed in animal models of atherosclerosis, and the results of these are highly divergent, finding that atherosclerosis increases, decreases or has no effect on infarct size (Table 1). One explanation for this may be that most rodent models of atherosclerosis do not completely mimic the pathophysiology of the atherosclerotic coronary artery. For example, in mice, a major limitation is that atherosclerotic plaques tend to manifest most frequently in the aorta and proximal great vessels, and they are rarely, if ever, seen in the coronary vessels [206].

**Table 1:** Features of the main genetic mouse models of atherosclerosis [adapted from [41]]

Strain of mice	Lipid profile (compared to WT mice)	Systemic atheromas (upon high fat/cholesterol diet)	Coronary atheromas (upon high fat/cholesterol diet)	Infarct size	Advantages	Limitations
*ApoE*^*−/−*^	Very high VLDL (1565 mg/dL) High LDL (143 mg/dL) Low HDL (45 mg/dL) [163] TC: 1821 mg/dL TG: 107 mg/dL	Lesions present in aortic root and branches, the carotid artery, mesenteric artery, femoral arteries, renal, pulmonary arteries, valve sinus	CA atheromas develop only slightly, and only in the CA origins. CA mostly protected	6–8 weeks old, permanent MI, → 1 day after MI, increased infarct size to ~ 62% (compared to ~ 43%), 7 days after MI decreased EF and FS in male mice [237] 12–16 weeks old, 30 min Isch, 2 h Rep → no effect on infarct size (~ 50% in both groups) in male mice (C57BL/6 background) [16] 10–12 weeks old, Western diet, 7 days after surgery, permanent MI, → 10 months later, no effects on heart failure in male mice (C57BL/6 background) [164]	Hypercholesterolaemia and spontaneous lesions on a normal diet and extensive atherosclerosis on a Western diet	No plaque rupture or thrombus formation Altered inflammation—role in plaque development Most cholesterol in VLDL
ApoE*3-Leiden	Very high VLDL High LDL Similar HDL TC: 67.1 mmol/l TG: 3.5 mmol/l [199]	Lesions present in ascending aorta, aortic arch, descending aorta, abdominal aorta Renal artery branch points	CA lesions only visible at 4 months	10–12 weeks old, Western diet 4 weeks before surgery, 45 min Isch, → 8 weeks later, reduced infarct size (12%) compared to ApoE*3-Leiden mice on normal diet (IS 22%) in female mice (C57BL/6 background) [162]	Susceptible to atherosclerosis on a Western diet, respond to lipid-lowering treatments with statins, fibrates, and niacin as humans Functional APOE—no effect on inflammation	No plaque rupture or thrombus formation
*LDLR*^−/−^	High VLDL (176 mg/dL) Very high LDL (484 mg/dL) Similar HDL (108 mg/dL) TC: 768 mg/dL [28]	Lesions present in aortic arch, brachiocephalic artery, thoracic aorta, abdominal aorta Gastrocnemius skeletal muscle arterioles	CAs protected from lesions	High cholesterol diet (HCD) for 2 or 12 weeks, 30 min Isch, 2 h Rep → Increase in infarct size (50% compared to 42%) after 2 weeks of HCD, but reduction of infarct size (13.2% compared to 22.5%) after 12 weeks of HCD in male mice (C57BL/6 background) [55]	lipoprotein pattern similar to humans, with high levels of LDL without any changes to HDL levels	No plaque rupture or thrombus formation Do not respond very well to lipid-lowering drugs No contractile dysfunction seen in coronary arteries [39]
*ApoE*^−/−^;*LDLR*^−/−^	Similar to ApoE^−/−^	Similar distribution to ApoE^−/−^ but more advanced lesions	Lesions present in CA after 6–8 months high fat/cholesterol diet	Larger infarcts than in WT on normal chow. IPC remains effective—male and female mice (C57BL/6 background) [111]	CA lesions	Requires modified diet for CA lesions
*SRBI*^−/−^;*LDLR*^−/−^	High VLDL (126 mg/dL) High LDL (167 mg/dL) Very high HDL (323 mg/dL) TC: 617 mg/dL [28]	Increase in diet-induced atheromas	Lesions present in aortic sinus and CAs, with complete occlusion, spontaneous MI from 3.5 weeks, on atherogenic diet	Several atherogenic diets tested, MI not performed but spontaneous infarcts present—female mice (mixed C57BL/6:129 backgrounds)	CA lesions, plaque rupture, thrombus formation	Spontaneous infarcts—variability in infarct size studies Infertile females
*SRBI*^−/−^;*ApoE*^−/−^		Aortic sinus from 4–7 weeks	Lesions present in aortic sinus and CAs, with complete occlusion,	Several atherogenic diets tested, MI not performed but spontaneous infarcts present—male and female mice (mixed C57BL/6:129 backgrounds)	CA lesions, plaque rupture, thrombus formation	Spontaneous MI even on chow diet

*CA* coronary artery, *EF* ejection fraction, *FS* fractional shortening, *HDL* high-density lipoproteins, *IPC* ischaemic preconditioning, *IS* infarct size, *Isch* ischaemia, *LAD* left anterior descending, *LDL* low-density lipoproteins, *Rep* reperfusion, *TC* total cholesterol, *TG* triglycerides, *MI* myocardial infarction, *VLDL* very low-density lipoproteins, *WT* wild-type

Mice are the most widely used species for reasons of cost, convenience, and their ability to be genetically manipulated. In this review, we examine the main mouse models of atherosclerosis currently available, their method of creation, pathology, coronary arterial function and response to cardiac IR. The aim is to identify which, if any, mouse model of atherosclerosis is suitable for investigating new cardioprotective drugs and strategies that will be clinically translatable to humans.

In humans, the left anterior descending (LAD) artery supplies a large territory of the ventricular myocardium—therefore, LAD occlusion tends to lead to larger infarcts and worse prognoses [40, 97]. As the main cause of MI in patients is plaque rupture, animal models would ideally include this event. However, since the timing of plaque rupture, and the location and size of the ischaemic area that result are all unpredictable, this is not experimentally practical. In any case, in most rodent models of atherosclerosis, the plaques rarely rupture [15, 236]. Therefore, in rodent IR studies of MI, the left coronary artery (equivalent to the LAD in humans [18]) is ligated at a specific location for 30–40 min, followed by reperfusion, which leads to infarction of ~ 40–60% of the AAR [18]. This degree of injury provides sufficient potential to detect salvage of the remaining viable tissue by cardioprotective interventions. In some studies, a model of permanent coronary artery ligation (CAL) is used, although this does not reflect the clinical scenario in which the occluded vessel is opened to reperfuse the ischaemic area as soon as possible. It is also important to note that after a certain duration of ischaemia (45–90 min in rodents and pigs, depending on the extent of coronary collateral vessels), ~ 90% of the AAR will be infarcted, and therefore, no significant reduction in acute infarct size is possible [14, 181]. Since only limited data is available, we will discuss both studies using either acute, reperfused (IR) or chronic ischaemic (CAL) models of MI.

### How does atherosclerosis develop in humans?

**Fig. 1:**
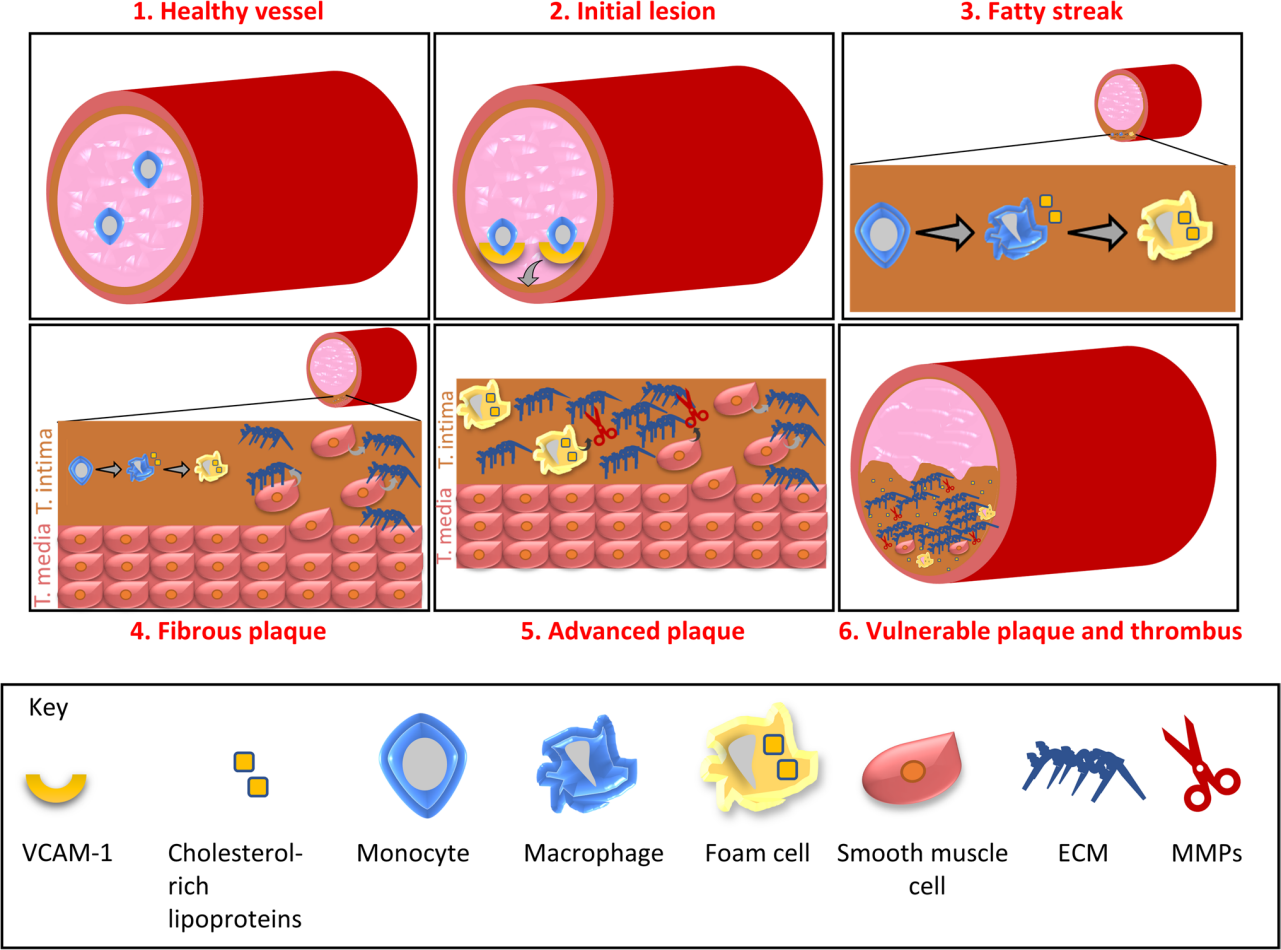
Process of atherosclerotic lesion development. **1** Monocytes circulate in the circulation in a healthy vessel. **2** Endothelial dysfunction leads to the expression of cell adhesion molecules such as VCAM-1 by activated endothelial cells. These cell adhesion molecules allow monocytes to adhere to the wall and infiltrate to the tunica intima. **3** Monocytes differentiate into macrophages and engulf cholesterol-rich lipoproteins, becoming foam cells. **4** Smooth muscle cells (SMCs) infiltrate and stimulate the production of extracellular matrix components. **5** Foam cells and smooth muscle cells release matrix degrading matrix metalloproteinases (MMPs). **6** Degradation of the extracellular matrix (ECM) by MMPs increases plaque vulnerability to rupture and thrombus formation

Atherosclerosis is a progressive inflammatory disease characterised by the accumulation of oxidized lipids in the arterial wall leading to the development of atherosclerotic lesions (Fig. 1). These lesions gradually harden and cause narrowing of the arterial lumen. They can remain stable for many years, but can eventually become large enough to impede the flow of blood through the vessel lumen to cause ischaemia. The major consequences of acute or chronic obstruction are MI, stroke, or peripheral artery disease, depending on the affected artery. Atherosclerotic plaques leading to acute coronary events tend to occur in the left anterior descending coronary artery, right coronary artery and the left circumflex arteries, and cluster within the proximal third of the vessel [54, 216]. Here, plaque rupture can initiate thrombosis formation, causing acute MI.

Several risk factors can modify the extent and rate of atherosclerosis development, including hyperlipidaemia, smoking, sex, diabetes and hypertension [230]. A key player in the development of atherosclerosis is an increase in plasma levels of cholesterol-rich lipoproteins such as low-density lipoprotein (LDL) [118, 146, 189]. Many studies have shown that oxidised LDL particles promote the progression of atherosclerosis [134, 142]. Endothelial dysfunction in the artery causes modification of the APOB-containing LDL and triggers the infiltration of monocytes into the artery wall. Due to the impaired function of the endothelium, LDL particles can deposit in the arterial wall and are retained by the extracellular matrix [17, 116]. LDL particles accumulate and bind to intimal proteoglycans, forming aggregates. These can then enter smooth muscle cells via LDL receptors [121]. Activation of the vascular endothelial cells also leads to the expression of cell adhesion molecules, such as VCAM-1 and ICAM-1, which facilitate the binding and adherence of immune cells such as monocytes to the arterial wall [82, 147]. These monocytes differentiate into macrophages. As cholesterol levels rise, the expression of the high-affinity LDL receptor drops but expression of the high-affinity scavenger receptors for LDL particles does not. These scavenger receptors allow the loading of macrophages with the excess cholesterol, forming foam cells and triggering inflammation [13, 49, 50, 136]. Importantly, in terms of investigating cardioprotective strategies, myocardial-resident macrophages have been associated with both injury [229] and repair [35] in response to MI. Foam cells can induce further inflammation by releasing cytokines such as interleukin-1β (IL-1β) and tumour necrosis factor-α (TNF-α) [117, 131]. Activated smooth muscle cells release extracellular matrix proteins such as collagen and elastin, which promote the formation of the fibrous cap [144]. Foam cells and extracellular lipid particles build up under the fibrous cap generating the necrotic core. As the atherosclerotic plaque progresses, the fibrous cap becomes more prone to rupture and releases its contents into the blood. This triggers thrombus formation and can lead to MI or stroke [67].

High-density lipoprotein (HDL), APOE and APOA-I counteract lesion development by promoting cholesterol efflux from peripheral tissues and preventing inflammation [65, 165, 213]. Nonetheless, inflammatory cytokines facilitate the infiltration and proliferation of smooth muscle cells into the lesion, which produce the extracellular matrix (ECM) forming a fibrous layer. Foam cells, and to a lesser extent smooth muscle cells and endothelial cells, release matrix-degrading metalloproteinases (MMPs). These can degrade all components of the ECM [48, 72, 224]. The degradation of the ECM increases the vulnerability of plaques to rupture. Plaque rupture leads to thrombus formation with aggregated platelets, blood coagulation and blockage of blood flow. The consequence of this occurring in a coronary artery is an acute MI.

Atherosclerosis is a chronic inflammatory disease, accompanied by innate and adaptive immune responses [221]. LDL and oxidised LDL act as self-antigens, stimulating CD4^+^ T cells, and driving an autoimmune response in atherosclerotic lesions fuelling plaque inflammation [98, 191]. CD4^+^ T cells can differentiate into T-helper cells that induce B cells to produce high-affinity IgG antibodies against LDL, oxidised LDL and APOB. These antibodies can either be atheroprotective or contribute to the progression of disease [221]. In patients with atherosclerosis, APOB-specific regulatory T cells appear to be atheroprotective by inhibiting atherogenic T-cell subsets and suppressing inflammation [5]. Nonetheless, as atheromas develop, this initial protective immune response promotes endothelial dysfunction, formation of foam cells and cell death via secretion of interferon-γ (IFN-γ) [5]. Therefore, despite initial atheroprotective features, with its potential to be both pro- and anti-inflammatory, the adaptive immune system can become pathogenic. The balance between a pro-inflammatory or anti-inflammatory response is partially controlled by genetics, which is relevant when considering the mouse models described below, which typically have low genetic diversity. Furthermore, differences in the immune system of mice and humans renders the direction and amplitude of autoimmunity in humans difficult to predict from mouse models [221].

The “response-to-injury” hypothesis of atherogenesis as defined by Ross proposes that “injury” to the endothelium is the initiating event in atherogenesis [175]. Furthermore, since the CANTOS trial, the involvement of inflammation in atherosclerosis is now firmly established [169]. Each of these aspects (lipids, endothelial function, chronic inflammation) could potentially influence MI. Therefore, the ideal experimental model of atherosclerosis for use in investigating cardioprotective strategies would entail all three aspects.

### Coronary endothelial dysfunction precedes atherosclerosis

Impaired endothelial function and arterial vasomotion can be observed prior to the arterial stiffening and remodelling that occurs as atherosclerosis proceeds. For example, epicardial and microvascular coronary endothelial dysfunction independently predict acute cardiovascular events in patients with coronary artery disease (CAD) [66]. Whereas acetylcholine causes vasodilation of healthy arteries, it induces paradoxical vasoconstriction in atherosclerotic coronary arteries with advanced stenosis and even in many arteries with minimal disease [123]. In contrast, smooth muscle function is less affected by atherosclerosis [123].

Nitric oxide (NO) is a key mediator of vascular homeostasis, modulating smooth muscle proliferation, inflammation, platelet activation, and vascular tone, each of which can impact the development of atherosclerosis. A major source of NO is nitric oxide synthase (NOS) in the endothelium. In healthy vasculature, endothelial NO suppresses the development of atherosclerosis by inhibiting platelet aggregation, inhibiting LDL oxidation, preventing infiltration of leukocytes into the vascular wall and inhibiting smooth muscle cell proliferation and constriction [46, 113, 114]. Importantly, however, endothelial NOS is impaired by atherosclerosis, and it can even produce damaging superoxide instead. Therefore, as with the adaptive immune system, an increase in NO can be either protective or deleterious in the setting of atherosclerosis, depending on its levels and duration [60, 195].

In a substantial number of STEMI patients in whom epicardial coronary artery reperfusion is achieved, reperfusion at the myocardial tissue level remains incomplete [145]. This is referred to as “no reflow” and is due to microvascular obstruction (MVO). As a consequence, despite apparent successful epicardial recanalization, the myocardium remains ischaemic, and will become infarcted [14]. Although MVO is independently associated with adverse ventricular remodelling and patient prognosis, MVO is not currently routinely measured or treated in patients. Contributing factors to MVO include coronary microvascular dysfunction and vasoconstriction, and physical obstruction of the microvessels by damaged endothelial cells or micro-embolization of atherosclerotic debris [75]. This provides a strong rationale for the use of animal models with coronary artery atherosclerosis, as this may affect the outcome of IR and cardioprotection studies.

Atherosclerosis sufficient to cause stenosis and ischaemia gradually affects the entire structure of the coronary tree by stimulating the development of coronary collateral vessels, such that they are detected in up to ~ 70% of patients with acute MI [183, 215]. This is important because residual blood flow carried by collaterals at the time of acute MI can limit infarct size.

In mouse studies, the aorta is commonly used to assess vascular function because its size makes it easier to study than other arteries [4, 9, 177]. This is not ideal for the study of atherosclerosis, however, since its first symptoms are not usually caused by plaques in the aorta, but rather the obstruction of flow through conduit arteries supplying major organs such as the heart or brain. Furthermore, both endothelium-dependent and -independent vasodilation differ significantly in magnitude between the aorta and other arterial segments from carotid, femoral, mesenteric, renal and coronary arteries [99]. The aorta is, therefore, not a good surrogate to study vascular function in other arteries.

## Mouse models of atherosclerosis

Most animal models of atherosclerosis are based either on feeding with a diet enriched in fat and cholesterol, or the introduction of genetic modifications known to alter cholesterol metabolism.

## Diet-induced models of atherosclerosis

The first animal model of atherosclerosis was developed in rabbits by feeding them a diet enriched in animal proteins (milk, meat and eggs), which resulted in lesions with a build-up of foam cells in the aorta [81]. Since that time, diets containing differing concentrations of fat, cholesterol and cholate have been widely used to induce atherosclerosis in animal models [53, 225]. Cholate, a bile acid that facilitates the digestion and absorption of lipids in the small intestine, has been used extensively to induce atherosclerosis, but is not ideal as it may cause nonspecific toxicity [47, 108, 109, 210, 211].

Vesselinovitch et al. developed the first atherosclerotic mouse model using a diet consisting of 30% fat, 5% cholesterol, and 2% cholic acid; however, this severe diet also resulted in weight loss and respiratory infections [211]. Paigen et al*.* found that when they reduced the fat content of the diet to 15%, C57BL/6 mice developed atherosclerosis more slowly, with lesions with fatty deposits and foam cells in the aorta appearing by 14 weeks [153]. Of note, the lesions were largely confined to the aortic root and did not develop further than a fatty streak [153]. To achieve plaques resembling human intermediate lesions and beyond, other models are required.

### Infarct size

Two weeks of high fat and cholesterol diet prior to surgery had no impact on infarct size in C57BL/6 mice subject to 30 min LAD ligation and 2 h reperfusion [55]. Surprisingly, however, after 12 weeks of high fat and cholesterol diet, infarct size after IR was significantly smaller than those on a normal diet [55]. In a separate study, a C57BL/6 J sub strain called C57BL/6JBomTac were fed an obesogenic diet containing 60% fat for 33 weeks and their hearts were isolated and subject to 30 min of global ischaemia and 60 min of reperfusion in a Langendorff model [37]. Infarct size was reduced in mice that were on the obesogenic diet, compared to those on a normal diet [37]. This could be due to an increase of the signalling substance sphingosine-1-phosphate (S1P) in obesogenic animals [74, 193]. S1P mediates endothelial barrier tightening [29, 180, 223] and may diminish the endothelial leakage with interstitial oedema formation, which is a major component of IR injury [74, 193].

Even though wild-type mice exhibit signs of dyslipidaemia upon a Western diet, their complicated phenotype must be considered when being used as a model of atherosclerosis. Besides hyperlipidaemia, these mice exhibit hyperglycaemia, corresponding to a diabetes mellitus type II phenotype. Therefore, their cardiac function and response to I/R and cardioprotective strategies can be potentially affected by the multi-faceted metabolic syndrome they exhibit [6].

### Limitations of the model

These early models helped to establish the role of cholesterol in atherogenesis, but are typically obesogenic, which is a potential confounding factor. Furthermore, the non-physiological nature and potential toxicity of cholate-containing diets suggest that atherogenesis in these mice may not accurately reflect the human disease [210].

## Transgenic mouse models of atherosclerosis

A number of genes with a critical role in the development and progression of atherosclerosis have been identified, and these have been exploited for the development of transgenic mouse models with enhanced atherogenesis. However, each model has its advantages and limitations which will be discussed.

## *ApoE* knockout mice

### Model

Apolipoprotein E (APOE) is involved in lipoprotein metabolism and lipoprotein-mediated lipid transport [7, 105]. It associates with plasma lipoproteins and plays a major role in their production, conversion and clearance from the blood [90, 105, 209]. The mouse APOE protein is ~ 70% homologous to the human protein [158, 161]. APOE is carried by chylomicrons and VLDL in plasma and acts as a ligand to mediate the uptake of these remnants by LDL receptor on the surface of hepatic cells, which remove them from the circulation [127, 205]. Knockout mice lacking APOE were developed in 1992, bringing the first mouse genetic model of atherosclerosis into existence [158, 161]. *ApoE* knockout mice (*ApoE*^−/−^) mice display delayed lipoprotein clearance and develop dyslipoproteinemia, hypercholesterolemia and atherosclerotic lesions even when fed normal chow [214].

### Lipid profile and cardiac and peripheral atheromas

*ApoE*^−/−^ mice on a normal chow diet have significantly increased levels of total plasma cholesterol in comparison to wild-type mice [140]. In humans, plasma cholesterol levels less than 200 mg/dL are considered healthy, whereas > 240 mg/dL is considered high. In wild-type mice on a chow diet total plasma cholesterol levels are only 75–110 mg/dL, but in *ApoE*^−/−^ mice they are dramatically increased to 400–600 mg/dL [140]. Even if this level is much higher than in patients, the fact that hypercholesterolemia and lesions develop spontaneously on a normal chow diet makes *ApoE*^−/−^ mice favourable to diet-induced models. In the plasma, APOE is associated with chylomicron remnants and VLDL. After binding to the LDL receptor with high affinity, APOE plays a key role in the clearance of VLDL and remnant proteins [166]. In comparison to wild-type mice, loss of APOE decreases levels of HDL and increases VLDL and LDL cholesterol levels [77, 87]. On a normal chow diet, lesions appear by 6 weeks of age. The presence of foam cells and smooth muscle cells in the lesion is observed at 8–10 weeks and fibrous plaques appear at 15 weeks. A Western-type diet can accelerate the growth and extent of the lesions depending on the requirements of the study. In *ApoE*^−/−^ mice, the pattern of lesion distribution in the heart is very different from that of humans. The lesions in these mice tend to form throughout the vasculature including in the aortic root and branches, the carotid artery, mesenteric artery, renal and pulmonary arteries as well as the valve sinus [27, 140]. The major lesions are located in the valve sinus, including the origins of the coronary arteries, but the lesions extend only a short distance onto the arterial trunks [80, 148]. Consequently, unlike in humans, the first segment and first branch of all the major coronary arteries are protected from disease [80, 148]. Despite the lack of robust atherosclerosis in coronary arteries of these mice, the lesions are a good model for the developmental process of the human disease in non-cardiac arteries. Interestingly, it has recently been shown transverse aortic constriction (TAC) performed in in *ApoE*^−/−^ mice leads to coronary plaque formation, progression, and myocardial events [129]. Furthermore, in several of the TAC-induced *ApoE*^−/−^ mice, evidence of myocardial infarction caused by embolism was obtained [129], which suggests the model could be used for studying no reflow caused by microemboli.

In regards to peripheral atheromas in *ApoE*^−/−^ mice, studies of the skeletal muscle of these mice demonstrate decreased capillary density from 12 weeks. Levels of nitric oxide, one of the key players in skeletal muscle function and metabolism [168], decline from the age of 20 weeks [187, 188]. Interestingly, isolated, Langendorff-perfused *ApoE*^−/−^ hearts displayed reduced nitric oxide-dependent vasodilation at the level of coronary resistance vessels [56]. In addition to extensive plaque formation in the aorta, plaques were observed in the femoral arteries of *ApoE*^−/−^ mice at the age of 65 weeks [11]. In *ApoE*^−/−^ mice, an increase in inflammation and a higher level of hydrogen peroxide in skeletal muscle were observed when compared with wild-type mice suggesting a protective role for APOE in peripheral arteries [159].

### Infarct size

In 12–16 week old *ApoE*^−/−^ mice, there was no difference between the area at risk or infarct sizes following 30 min regional ischaemia of the myocardium followed by 2 h reperfusion, compared to wild-type mice [16] (Table 1). The impact of APOE deficiency on MI-induced heart failure was investigated by performing chronic LAD ligation in 6–8 week old *ApoE*^−/−^ mice. One day following permanent CAL, infarct size and myocardial injury were significantly greater in *ApoE*^−/−^ mice compared to wild type, as assessed by TTC staining, cTnI and CK-MB [237]. In contrast, in a separate study in which mice were fed a Western diet from the 7^th^ day following permanent CAL, no effects of APOE deficiency were observed on infarct size, survival or the development of heart failure over the following 10 months [164]. Infarct size was also similar between wild type and *ApoE*^−/−^ mice when using an IR model, which consisted of 30 min ischaemia and 2 h reperfusion [38]. Only one study has addressed whether cardioprotection remains effective in *ApoE*^−/−^. This showed that saffron aqueous extract reduces myocardial infarct size in both *ApoE*^−/−^ and wild-type mice to a similar extent [38]. Though *ApoE*^−/−^ mice have been extensively studied, it is surprising to find only one study on cardioprotective strategies in this strain. Further research of cardioprotection in *ApoE*^−/−^ mice would be informative and aid the translation of therapeutic strategies into patients with atherosclerosis.

### Limitations of the model

There are several caveats to using this model for studies of MI. Thrombotic occlusion rarely occurs in the coronary arteries of *ApoE*^−/−^ mice, unlike in humans, making it more difficult to extrapolate findings into the clinic, as the effect of atherosclerosis on MI could be different to that which occurs in humans. The low prevalence of plaque rupture in mice could potentially be due to the small diameter and the lower surface tension of the mouse vessel in comparison to humans [88]. Nonetheless, the plaques in the brachiocephalic artery of older (42–60 weeks) *ApoE*^−/−^ mice are more similar to human plaques, involving intra plaque haemorrhage, an acellular necrotic core and erosion of the necrotic mass into the lumen [173]. In a study of *ApoE*^−/−^ mice after 8 weeks of fat feeding, plaque rupture was observed in the brachiocephalic arteries at high frequency [93]. Plaque rupture has been extensively studied in *ApoE*^−/−^ mice and it appears that brachiocephalic arteries and the aorta are the main arteries where plaque rupture occurs in this model [24, 93, 219]. Moreover, APOE has an impact on inflammation, oxidation, reverse cholesterol transport by macrophages and proliferation and migration of smooth muscle cells from tunica media in the vessel wall into the tunica intima, which could play a role in plaque development in *ApoE*^−/−^ mice regardless of changes in the lipid profile [52]. Following MI, Ly-6C^hi^ monocytes digest damaged tissue in the first 4 days and Ly-6C^lo^ monocytes facilitate angiogenesis and repair of the tissue from days 5–10 [139]. Inflammatory gene expression and protease levels associated with the activity of Ly-6C^hi^ monocytes, were more pronounced in *ApoE*^−/−^ mice 5 days after MI [154]. APOE is a multifunctional protein with roles in inflammation, oxidation, smooth muscle proliferation and migration as well as reverse cholesterol transport by macrophages. Thus, changes in these functions may affect the development of atheromas in *ApoE*^−/−^ mice, independent of their changes in lipoprotein profile [52]. Another limitation to be considered with *ApoE*^−/−^ mice is the high levels of VLDL particles, which is not typical in human atherosclerosis [163].

## ApoE*3-Leiden mice

### Model

In an uncommon disorder of lipoprotein metabolism called familial dysbetalipoproteinemia (FD), the *ApoE* gene is mutated, which results in reduced binding to receptors and decreased clearance of the remnants. The accumulation of these particles results in hypercholesterolemia and hypertriglyceridemia, predisposing FD patients to coronary artery disease and peripheral artery disease. ApoE*3-Leiden, a mouse model of FD, contains the human *APOE3Leiden* and *APOC1* genes [199]. APOC1 inhibits lipoprotein lipase (LPL) which is involved in the lipolysis of triglyceride-rich lipoproteins [107]. As in the human disease, the mutation in the *ApoE* gene results in a dysfunctional protein with reduced binding to its receptors impairing the clearance of chylomicron remnants and VLDL [128, 220]. Consequently, these mice are susceptible to atherosclerosis when fed a high-fat diet. In contrast to other mouse models of atherosclerosis, they respond to lipid-lowering treatments such as statins, fibrates, and niacin in a similar way to humans [8, 102, 198, 201], which has made them a useful experimental model for drug development.

### Lipid profile and cardiac and peripheral atheromas

**Fig. 2:**
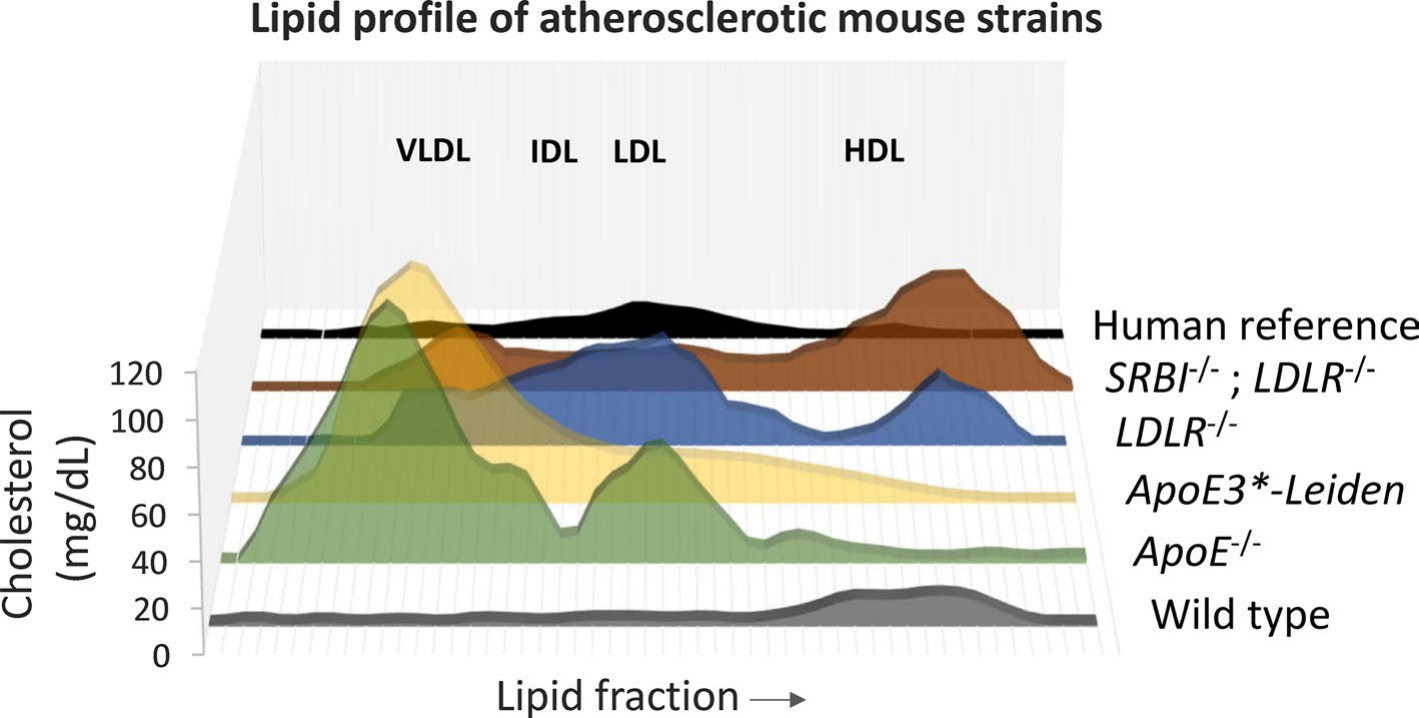
Lipid profile of different knockout mouse models of atherosclerosis. Unlike humans, wild-type mice carry cholesterol primarily in HDL particles. In comparison to human, *ApoE*^−/−^ and ApoE3*-Leiden mice have very high levels of VLDL cholesterol, higher levels of LDL cholesterol and similar levels of HDL cholesterol. *LDLR*^−/−^ mice have higher levels of all cholesterol-loaded lipoproteins and *SRBI*^−/−^; *LDLR*^−/−^ mice have higher levels of VLDL cholesterol, similar levels of LDL cholesterol and much higher levels of HDL cholesterol than humans [adapted from [28, 83, 163, 199]]

On several diets tested, ApoE*3-Leiden mice developed enhanced aortic atherosclerosis compared to wild-type mice [63]. Total plasma cholesterol levels were significantly higher in ApoE*3-Leiden mice on both normal chow and high-fat diet [63]. The lipoprotein pattern on a high-fat diet was characterised by increased levels of VLDL and LDL and decreased levels of HDL (Fig. 2) [199]. Early lesions in ApoE*3-Leiden mice included those on aortic valves and the free aortic wall, which were superficial and contained one or two layers of lipid-loaded foam cells. Some of these lesions developed into more extensive plaques covering the entire arterial wall, and were rich in foam cells. With a more severe diet such as high fat and cholate for 3 months, more complex lesions developed, containing a lipid-laden core loaded with foam cells with calcification in certain sections [111]. Lesions were present in the ascending aorta, aortic arch, and descending aorta, whereas wild-type mice showed no lesions when fed the same diet [63]. However, none of the animals had lesions in the proximal coronary arteries [63]. In another study in which ApoE*3-Leiden mice were fed a special high-fat and cholesterol diet (15% cacao butter, 0.5% cholate, 1% cholesterol, 40.5% sucrose, 10% corn starch, 1% corn oil, and 4.7% cellulose), extensive atherosclerosis was observed [124]. After 4 months, advanced lesions were present in the aortic root, the aortic arch and its main branch points. Plaques were also present in the abdominal aorta and renal artery branch points [124]. Early lesions (fatty streaks) were observed in the proximal coronary arteries, and these had become advanced by 6 months [124]. After 12 months, calcifications were seen in the coronary arteries of some mice.

The major differences between humans and ApoE*3-Leiden mice are the lack of plaque rupture, thrombus formation and haemorrhage in these mice [174]. The possible reasons for this could be due to the morphologically intact endothelial layer, which prevents rupture, and the low level of apoptosis in the fibrous cap.

### Infarct size

There are few infarct studies in ApoE*3-Leiden mice to date, even though these mice appear to be a suitable animal model of atherosclerosis [199]. Nonetheless, one study has investigated the effect of 4 week diet-induced hypercholesterolemia on left ventricle (LV) remodelling following IR in these mice in comparison to normocholesterolemic ApoE*3-Leiden mice. Surprisingly, 8 weeks following IR, a significant reduction in infarct size and an increase in wall thickness were observed in hypercholesterolemic mice, with less accumulation of infiltrated inflammatory cells [162]. Paradoxically, LV contractile function was significantly impaired in hypercholesterolemic ApoE*3-Leiden mice, reflected by a reduction in LV ESP, dP/dtMAX, and dP/dtMIN, though no differences of LV dimensions were observed [162].

### Limitations of the model

One limitation of the ApoE*3-Leiden model is similar to the other mouse models of atherosclerosis in that thrombosis does not occur. Therefore, it is difficult to relate the data obtained from this strain to the clinic. In addition, lesions are not observed in the coronary arteries [63], or are only observed after a relatively long time of 6 months of high fat and cholesterol diet [124].

## *LDLR* knockout mice

### Model

The LDL receptor (LDLR*)* is a glycoprotein expressed on the surface of hepatocytes, which plays a key role in the endocytosis and removal of circulating LDL cholesterol. Besides the widely used *ApoE*^−/−^ mice, *LDLR*^−/−^ mice are one of the most popular choices amongst mouse models of atherosclerosis. Mutations in the *LDLR* gene in humans cause familial hypercholesterolemia, with elevated plasma LDL cholesterol levels and deposition of cholesterol in vessels leading to the formation of atherosclerotic plaques [23, 110]. Plasma cholesterol is moderately elevated in *LDLR*^−/−^ mice fed normal chow and they develop atherosclerosis gradually, although this can be accelerated on a high-fat diet [101, 137, 182]. The lipoprotein profile in the *LDLR*^−/−^ mice on chow diet is similar to humans, with high levels of LDL (Fig. 2) [84].

### Lipid profile and cardiac and peripheral atheromas

The types and locations of lesions in *LDLR*^−/−^ mice are similar to *ApoE*^−/−^ mice, and build up gradually with the earliest lesions seen in the proximal aorta. In general, the *LDLR*^−/−^ model is a milder model of atherosclerosis than the *ApoE*^−/−^ mice mainly due to the lower hyperlipidaemia observed [21, 184]. *LDLR*^−/−^ mice develop mild or no lesions when on a normal chow diet. Nonetheless, on a Paigen diet, the development of atherosclerosis is accelerated with significant increases in cholesterol levels and size of atherosclerotic plaques [85]. High-fat diet induced the development of atherosclerotic plaques with fatty streaks and accumulation of foam cells in the early lesions in the aortic root after 5 weeks [235]. Early lesions were also observed in the aortic arch and brachiocephalic artery and increased in size until they occupied most of the surface [125]. After one month on a high-fat diet, the tunica media had thickened significantly along with the appearance of lipid-laden macrophages. After 9 months, advanced lesions caused narrowing of the brachiocephalic artery. Surprisingly, lesions in the thoracic aorta were only visible after 6 months of high-fat diet, and abdominal aorta lesions were only detected after 9 months, although they increased significantly after this time point [125]. These changes in the rate of development for lesions in different regions, results in variable sensitivity to treatments that alter atherosclerosis. In terms of its effect on the peripheral circulation, LDLR loss leads to a decrease in capillary density in the gastrocnemius muscle by the age of 22 weeks, and the ratio of wall to lumen in skeletal muscle arterioles is elevated [187]. Importantly, no evidence of endothelial dysfunction was seen in functional studies of mesenteric artery, coronary artery or aorta of LDLR^−/−^ fed either normal chow or Western diet for 8 weeks [39].

*LDLR*^−/−^ mice have been crossed with *ApoE*^−/−^ mice to generate *ApoE*^−/−^; *LDLR*^−/−^ double knockout mice. These have a similar lipid profile to *ApoE*^−/−^ mice, with a marked increase in VLDL and chylomicron remnants [86]. After 6–8 months of a high-fat and cholesterol diet, the *ApoE*^−/−^; *LDLR*^−/−^ develop extensive atherosclerotic lesions throughout the coronary tree [112]. Interestingly, the vasoconstrictor response to endothelin-1 (ET-1) was enhanced in thoracic aortic rings of *ApoE*^−/−^; *LDLR*^−/−^ mice in comparison to wild-type mice [91]. However, endothelial-dependent acetylcholine-induced relaxation was significantly impaired [91]. The combined administration of L-arginine and BH(4) reversed the endothelial dysfunction of the *ApoE*^−/−^; *LDLR*^−/−^ mice [91].

### Infarct size

Interestingly, after 30 min LAD ligation and reperfusion for 2 h, infarct size in *LDLR*^−/−^ mice was roughly half that seen in wild type [55]. After feeding *LDLR*^−/−^ mice a high cholesterol diet for 12 weeks, infarct size following IR was further reduced to a significant degree, leading the authors to suggest that prolonged exposure to high levels of plasma cholesterol protects the myocardium from IR injury [55]. This is surprising since it contrasts with the majority of previous studies on hypercholesterolemia in other animal models [58, 150, 212]. However, the interpretation of this data is complicated since in the same study, a shorter, 2 week diet caused a dramatic doubling of infarct size following IR [55]. The authors suggested that this may be due in part to the extremely high levels (> 2000 mg/dL) of cholesterol that are obtained in *LDLR*^−/−^ mice following the high-fat diet.

Hearts were isolated from *ApoE*^−/−^; *LDLR*^−/−^ mice on a high-fat and cholesterol diet, and Langendorff perfused. Following 40 min global ischaemia and 60 min reperfusion, *ApoE*^−/−^; *LDLR*^−/−^ hearts had larger infarcts, more troponin T release, and worse cardiac function than wild-type mice [112]. However, because the wild type mice were fed normal chow, the difference could be due either to hypercholesterolemia or atherosclerosis. Interestingly, ischaemic preconditioning (IPC) remained equally effective in both *ApoE*^−/−^; *LDLR*^−/−^ and wild type hearts [112].

### Limitations of the model

Although it is a widely used model of atherosclerosis, *LDLR*^−/−^ mice do not respond very well to lipid-lowering drugs used in patients, indicating possible pathophysiological differences compared to human disease [8, 231]. In addition, the discrepancy between humans and mouse models in general such as the lack of plaque rupture applies to the *LDLR*^−/−^ model. The combined *ApoE*^−/−^; *LDLR*^−/−^ model may be closer to the cardiac patients with coronary atheromas.

## *SRBI* knockout mice

### Model

Scavenger receptor class B, type I (SRBI) is an HDL receptor expressed on the surface of multiple cell types and mediates the selective uptake of plasma HDL cholesterol by the liver. Hence, it regulates HDL cholesterol levels, particle size, and metabolism of cholesterol by steroidogenic tissues [2, 104, 179]. HDL cholesterol levels inversely correlate with the risk of atherosclerosis, most probably due to the role SRBI plays in reverse cholesterol transport [59, 94, 192]. Excess cholesterol from peripheral tissues is removed and delivered to the liver from where it is either redistributed to tissues in which it is required, or removed by the gall bladder. HDL cholesterol content is pivotal to the development of atherosclerosis as it also plays a role in inflammation [12, 143].

The extracellular domain of human SRBI shares 80% sequence identity with the mouse protein. However, unlike mice, human hepatocytes express a second HDL-cholesterol receptor called cholesteryl ester transfer protein (CETP) [79, 178, 208]. Human SRBI has a similar function to the mouse protein and a similar pattern of expression [1, 106, 138]. In endothelial cells, SRBI is functionally involved in NO production resulting in the attenuation of monocyte adhesion [64]. In addition, HDL binding to SRBI on platelets inhibits aggregation and increases platelet survival [79, 178, 208]. One important point for studies involving *SRBI*^−/−^ mice is that deficiency of SRBI in female mice can cause infertility due to defects in oocyte development [133].

SRBI is a key regulator of HDL cholesterol levels and its overexpression leads to an increase in biliary cholesterol content in support of gene-targeting studies that suggest SRBI plays a key role in reverse cholesterol transport [170, 207, 217]. Moreover, SRBI binds LDL and VLDL to promote the efflux of un-esterified cholesterol from cells to HDL [89, 186]. Inactivation of SRBI leads to decreased reverse cholesterol transport and increased plasma HDL cholesterol levels [170, 207]. SRBI deficiency does not affect hepatic cholesterol levels or key regulators of hepatic cholesterol homeostasis, such as HMG-CoA reductase, the low-density lipoprotein receptor, or cholesterol 7α-hydroxylase (78).

### Lipid profile and cardiac and peripheral atheromas

Several groups have used *SRBI*^−/−^ mouse models to investigate the function of SRBI. On a chow diet, SRBI deficiency caused an increase in total serum cholesterol levels as a result of higher levels free cholesterol and an increase in the cholesterol carried in the HDL particles (Fig. 2) [203]. On a high-cholesterol Western diet for 20 weeks, *SRBI*^−/−^ mice developed more atherosclerotic lesions at the aortic root in comparison to wild-type controls [203].

*SRBI*^−/−^ mice have been crossed with other knockout strains to exacerbate the phenotype. Knockout of *SRBI* in either *ApoE*^−/−^ mice or *LDLR*^−/−^ mice causes an increase in larger HDL particles, highlighting the role of SRBI in reverse cholesterol transport [28, 197, 203]. Absence of *SRBI* in *LDLR*^−/−^ mice lead to a sixfold increase in diet-induced atherosclerosis [28]. No signs of coronary artery atheroma or ischaemic heart disease were identified when mice were fed a standard Western diet [115]. However, following 12 weeks on a modified Western diet containing higher (0.5%) cholesterol, coronary artery atherosclerosis was observed, being most severe at the aortic sinus level. The mice also developed spontaneous cardiac ischaemia/infarction systolic dysfunction and LV dilatation and died by 20 weeks [115].

A study on the effects of different high-fat diets with and without cholate and cholesterol has reported an increase in aortic sinus plaque formation and size as well as reduced survival in *SRBI*^−/−^; *LDLR*^−/−^ mice in comparison to *LDLR*^−/−^ controls on all atherogenic diets tested [47]. Interestingly, all *SRBI*^−/−^; *LDLR*^−/−^ mice developed atherosclerosis in their coronary arteries when on atherogenic diets, though this burden did not correlate with plaque sizes in the aortic sinus. In contrast, mice fed normal chow developed little atherosclerosis in their coronary arteries by 22 weeks of age. Uniquely, the double knockout mice showed signs of thromboses, which stained for the platelet marker, CD41, in the coronary arteries [47].

In *SRBI*^−/−^; *ApoE*^−/−^ mice, substantial atherosclerotic plaques were observed in the aortic sinuses after 4–7-week-old on a chow diet, compared to absence of plaques in *ApoE*^−/−^ or *SRBI*^−/−^ mice [197]. Coronary artery atherosclerosis with complete coronary artery occlusion was observed in *SRBI*^−/−^; *ApoE*^−/−^ mice, and these mice spontaneously developed multiple MIs, cardiac dysfunction and death at 5–8 weeks when fed a chow diet [20]. Their coronary artery lesions were strikingly similar to human atherosclerotic plaques, with evidence of haemorrhage and clotting [20].

*SRBI*^−/−^ crossed with mice harbouring the hypomorphic *ApoE* allele *ApoE*^R61h/h^ display features of hyperlipidaemia upon a high-fat diet leading to coronary plaques, partially occlusive coronary stenoses, spontaneous MI and reduced survival, closely resembling human coronary artery disease [156, 233]. Most thrombi were in medium and large coronary arteries in the basal LV, which explains the septal location of the thrombotic coronary arteries, as septal arteries are direct proximal branches of the right or left coronary artery or the aortic sinus [44]. *SRBI*^−/−^; *ApoE*^R61h/h^ mice display several of the previously identified features of human vulnerable plaques [226], such as the presence of cholesterol-rich plaques in larger vessels and proximal segments of the coronary tree, thrombi with a necrotic core, perivascular inflammation, MIs and spontaneous deaths [73]. Interestingly, coronary vasodilator response was assessed in these mice by coronary angiogram, and an impairment of NO-mediated dilation of conductance and microvessels was seen [156].

### Infarct size

The involvement of SRBI in circulating cells in protection from atherosclerosis is demonstrated by studies in which restoration of *SRBI* expression in bone marrow-derived cells in knockout models results in attenuation of coronary artery atherosclerosis, MI and cardiac enlargement in *SRBI*^−/−^; *ApoE*^R61h/h^ mice [157]. All *SRBI*^−/−^ mice in this study exhibited coronary atherosclerosis, but increased heart weights and cardiac enlargement were observed only in a subset of mice. This suggests that the cardiac enlargement and fibrosis are the result of extensive coronary artery atherosclerosis in this model which appears to be reduced upon SRBI restoration, potentially through reduced levels of monocyte recruitment [157].

*SRBI*^−/−^; *LDLR*^−/−^ mice develop spontaneous infarcts as early as 3.5 weeks depending on the atherogenic diet [47]. Nonetheless, surgically-induced MIs have not yet been studied in any of the SRBI^−/−^ mice and whether the presence of coronary artery atheromas have an impact on cardioprotection is yet to be investigated.

## Additional considerations

In any mouse experiment, it is important to consider the genetic background of the strain of wild-type mice being used, as this can significantly affect the results obtained. A study of 16 different inbred mouse strains fed the Paigen diet for 14 weeks, found major differences in the extent of atherosclerotic lesions that formed in the aorta [152]. The commonly used strain C57BL/6 was one of the most susceptible, developing lesions by 7 weeks with large plaques in the aorta and coronary arteries, which continued to develop. Strain 129, a common background strain for transgenic studies, had smaller lesions, and other strains such as C3H, and CBA had no lesions at all after 14 weeks on the Paigen diet. Strains AKR and DBA/2 displayed fatty streaks or lesions by 7 weeks, but these did not grow in size [152]. Another point to consider is that the impact of co-morbidities such as atherosclerosis on endothelial function and myocardial infarction may be sex-specific [160, 196]. It is, therefore, important to use both male and female mice in studies.

Transgenic models of atherosclerosis can also be highly dependent on the background strain. For example, deletion of *ApoE* causes an increase in HDL-cholesterol and triglycerides, which results in atherosclerotic lesions at the aortic arch, but these develop earlier in 129/SvEv mice than in C57BL/6 [126]. On the other hand, the atherosclerotic plaques in the aortic root develop faster in C57BL/6 mice [126], possibly reflecting anatomical differences between the two strains. In any case, these results highlight the contribution made by the genetics of the background strain to the development of atherosclerosis [126].

Several genes have been identified that can modify atherosclerosis development, and mutation of these can improve the modelling of aspects of atherosclerosis lacking from standard models. For example, heterozygous mutation of fibrillin-1 (*Fbn1*) in *ApoE*^−/−^ mice leads to fragmentation of the elastic fibres in the vessel wall, resulting in a mouse model more prone to the formation of vulnerable plaques and plaque rupture [200, 204]. These mice also manifested leaky plaque neo-vessels and intra plaque haemorrhage, resulting in plaque rupture, myocardial infarction, stroke, and sudden death [200]. Therefore, this model could serve as a highly translational model of atherosclerosis and atheroma rupture, as observed in patients. Whether such a model would provide a practical advantage in experiments designed to investigate cardioprotection, where the duration and extent of ischaemic injury is typically carefully controlled and reproducible, remains to be established.

A possible limitation of genetic models is that the majority result in complete absence of the protein throughout the life of the mice, from embryogenesis to adulthood. Viral-mediated models have the advantage of allowing the induction of atherosclerosis in adults, more closely reflecting the human situation. For example, proprotein convertase subtilisin/kexin type 9 (PCSK9) is a serine protease with a key role in the degradation of LDLR in the liver [130, 232]. In humans, mutations in the *PCSK9* gene have been associated with hypercholesterolemia and CVD [141]. Adenoviral overexpression of PCSK9 in mice decreases LDLR levels, leading to increased plasma LDL levels [130]. Mice expressing a gain-of-function mutant of PCSK9 delivered by adeno-associated virus (AAV) and fed a Western diet, develop sustained hyperlipidaemia with an increase in LDL and VLDL levels [172]. Atherosclerotic lesions with vascular calcification were observed in the aortas within 15–20 weeks, similar to plaques in *LDLR*^−/−^ mice [57]. However, the phenotype, like in *LDLR*-/- mice, is a consequence of loss of LDLR expression, and so the model is similarly limited by the absence of plaque development in coronary arteries. Furthermore, the response appears to be highly strain dependent, with the strongest effect in C57BL/6, and PCSK9 may also have additional effects independent of the LDLR that may affect studies [30].

A further consideration in the use of mouse models is the methods used for the identification of atherosclerotic lesions. Plaques are usually visualised by ex vivo staining with Oil-Red Sudan IV or Van-Gilson staining [135]. Nonetheless, the ability of these techniques to identify small/early atherosclerotic lesions is limited, and more advanced imaging modalities such as ultrasound, PET or microCT might be better suited to evaluating atheroma progression in carotid arteries, aorta and even coronary circulation in mouse hearts [25, 190]. Therefore, the limitations in translational value of in vivo models could be overcome by using robust techniques for plaque identification and localisation.

## Other animal models

**Fig. 3:**
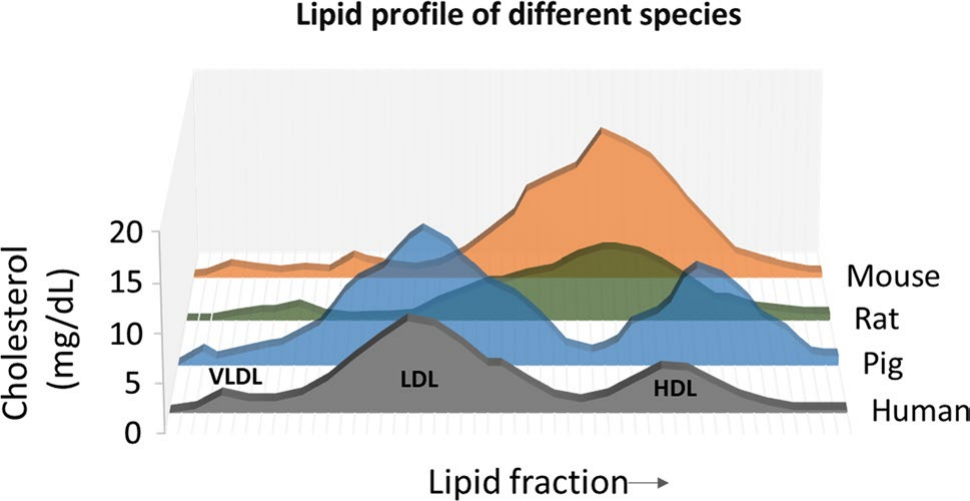
Lipid profile comparison across species. In contrast to human, most species carry cholesterol mainly in HDL particles. In mice and rats, LDL cholesterol is lower than HDL cholesterol. Rats have a strong overlap between both LDL and HDL particles. Pigs have a lipoprotein profile comparable to human characterized by high LDL cholesterol [adapted from [95]]

Given that few mouse models of atherosclerosis develop coronary artery plaques, it is worth asking the question of whether mice are the most suitable model for IR studies, or if other animal models are preferable. Rats have similar advantages to mice in terms of maintenance and cost. Nevertheless, due to the lack of a gall bladder, absorption of cholesterol is low in rats, and their lipid profile also differs substantially from that of humans (Fig. 3). In addition, it is difficult to induce plaque formation in rats by high-fat diet alone and most research in atherosclerotic rats combines the use of drugs and artificially induced endothelial injury [171, 228]. Genetic modification in rats became feasible only recently due to the optimisation of gene editing technologies [227, 234].

In contrast to rodent models, larger animals are more challenging to maintain, have higher associated costs, and take longer to develop atherosclerotic lesions. Nonetheless, rabbits have been a popular choice when it comes to models of atherosclerosis and have particular advantages. Their sensitivity to high-fat diets, high rate of cholesterol absorption, the ease of converting exogenous cholesterol into plasma lipids and low cholesterol clearance levels make rabbit an attractive model to study atherosclerosis. However, their herbivorous diet leads to major differences in lipid metabolism in comparison to humans and raises questions about the suitability. Plaques in rabbits have a typical lipid core covered by a fibrotic cap that can lead to MI, making them a relevant model of atherosclerosis [42]. Although anatomy of the heart and coronary circulation is similar to that of humans, atherosclerotic plaques mainly form in the thoracic aorta, and coronary lesions are usually restricted to the left coronary arterial trunks [78].

Pigs are susceptible to atherosclerosis upon hypercholesterolemia-induced by a high cholesterol diet, usually containing cholate to prevent the production of bile from cholesterol [3, 51]. A combination of high cholesterol diet and induced vascular injury is usually required to reduce the time for plaque development. Vascular injury can be achieved through methods such as percutaneous intramural injection of cholesteryl esters and human oxLDL, guidewire-induced injury, balloon inflation or partial vessel ligation [61, 62, 194]. In addition, pigs are anatomically and phylogenetically closer to humans compared to other models, and similarly are omnivorous. Finally, the size of the heart and coronary arteries makes them adequately suited to study atherosclerosis. Indeed, thrombus formation, plaque ruptures, IR injury and the fact that atherosclerosis develops in older pigs, provides a comparable platform to study the human disease and test therapeutic interventions that can be used in the clinic [10, 167, 185].

## Summary and conclusions

In the context of atherosclerosis, many studies utilize mouse models due to their small size, low associated costs and a plethora of available transgenic strains. Although several studies have investigated infarct size in these models, the efficacy of cardioprotective strategies have not yet been studied extensively. There are several factors to consider when choosing the ideal model as summarised in Table 1. The lack of coronary artery lesions impacting coronary artery function in most mouse models, make it difficult to be certain that therapeutic interventions can be translated into the clinic. In addition, the lipoprotein profiles and mechanisms of metabolism are an important aspect that affects disease progression and must also be considered in the chosen model. Of the most widely studied strains, *ApoE*^−/−^ mice and *LDLR*^−/−^ mice are popular choices for the study of atherosclerosis due to their susceptibility to developing atherosclerotic lesions. However, the atherosclerotic plaques are mainly located in the aorta and the surrounding regions, and no evidence of coronary artery dysfunction in these models has been reported. Despite their limitations, the transgenic mouse models reviewed here contribute to the overall corpus of knowledge on the effect that these genetic mutations have on cardioprotection in mouse models. Whether the effects observed are strictly due to atherosclerotic changes in the heart, or, as seems more likely, other functions of the proteins in the cardiovascular system, remains to be clearly determined. A better model for coronary artery atherosclerosis appears to involve *SRBI*. Though not without its caveats, *SRBI*^−/−^; *LDLR*^−/−^ mice provide a platform where coronary artery plaque development occurs on a normal diet in a relatively short time. This enables the study of IR and cardioprotection in a model that, it is hoped, more accurately reflects the response of the cardiac patient.

In conclusion, the huge number of cardioprotective compounds and treatments that have been shown over the past decades to be effective in mice, despite the lack of successful translation to animals, may be a sign of the bias inherent in the commonly used experimental model of mouse infarction. In order to discover cardioprotective strategies that are not only effective in mice but can be translated successfully to benefit STEMI patients, we suggest that it is important to use mouse models of atherosclerosis that exhibit lesions in the coronary arteries. Ideally, these lesions would result in vascular dysfunction, as is seen in patients, since this dysfunction is likely to have a major impact on the induction and effectiveness of certain cardioprotective modalities. In IR studies with infarct size as an endpoint, ischaemia is artificially induced by coronary ligation, so plaque vulnerability and rupture are not technically essential for the model. Similarly, it may not be crucially important that plaque morphology differs somewhat between mice and humans. On the other hand, if plaque rupture and micro-embolism *do* occur in the atherosclerotic mouse model, it might better mimic the contribution that MVO makes to IR injury. However, the level of spontaneous plaque rupture and myocardial infarction should not be excessive, or it could interfere with the measurement of the experimentally induced infarct. Although the study of atherosclerosis and coronary function in mouse coronary arteries is challenging, we believe these studies are necessary for the future development of ideal IR models in mice.
